# A Case of Schwannoma of the Submandibular Region

**DOI:** 10.2174/1874210601812010012

**Published:** 2018-01-29

**Authors:** Babatunde O. Bamgbose, Akiko Sato, Yoshinobu Yanagi, Miki Hisatomi, Yohei Takeshita, Irfan Sugianto, Junichi Asaumi

**Affiliations:** 1 Department of Oral and Maxillofacial Radiology, Okayama University, Graduate School of Medicine, Dentistry and Pharmaceutical Sciences, Okayama, Japan.; 2 Department of Dentomaxillofacial Radiology and Oral Diagnosis, Okayama University Hospital, Okayama, Japan.; 3 Oral Inspection and Diagnostic Center, Okayama University Hospital, Okayama, Japan.; 4 Dental Comprehensive Diagnosis Department, Okayama University Hospital, Okayama, Japan.

**Keywords:** Schwannoma, Submandibular region, MRI, Antoni type A, Antoni type B, Heterogeneous hypointensity

## Abstract

**Background:**

We herein described a rare case of schwannoma of the hypoglossal nerve in the submandibular region with diagnostic imaging and histopathological findings.

**Case Report:**

A 31-years-old woman has had a palpable firm, rubbery, freely mobile mass in the submandibular region. Of imaging, MR images showed homogeneous isointensity on T1-weighted imaging (T1-WI), heterogeneous hypointensity on T2-WI, heterogeneous hyperintensity on short T1 inversion recovery (STIR), and heterogeneous enhancement on contrast-enhanced T1-WI. A clear capsule was observed on the margin and showed hypointense on T2-WI. Dynamic MRI showed heterogeneous gradual increased enhancement. The uptake of contrast medium was regionally slow. Diagnostic imaging using CT and MRI was suspected of salivary gland tumor or neurogenic tumor. In consideration of imaging diagnosis, a pleomorphic adenoma or a schwannoma was suspected. Final diagnosis was confirmed on the basis of histopathological finding and intraoperative findings.

**Conclusion:**

1. Histopathologic examination is inevitable, because MR findings are not specific.

2. Schwannomas were said to have specific MRI properties, including specific signs (split-fat sign, fascicular sign, target sign). However, they are not always observed.

3. This case confirmed the differential diagnosis on the basis of the intraoperative finding that the tumor was continuous with the hypoglossal nerve.

## 1 INTRODUCTION

Schwannoma is a benign soft tissue tumor derived from Schwann cell of the peripheral nerve. Approximately 25 to 45% of extracranial schwannomas occur in the head and neck, most likely around the glossopharyngeal space and the carotid artery sheath. Although occurrence in other sites is relatively rare, it has been reported to occur in the parotid gland, nose, paranasal sinuses, and the oral cavity [1-3]. Schwannomas are usually unilocular, cystic, symptomless, slow-growing benign, solitary, encapsulated tumors that are attached or surrounded by a nerve. They appear to push the nerve axions and can often be dissected free, with preservation of the nerve of origin. Many schwannomas are symptomless and discovered incidentally on routine imaging. Sometimes, patients present with clinical symptoms including slow-growing, freely mobile, well delineated, localized lobular mass. Schwannomas most commonly arise in the soft tissues and intraosseous schwannomas are rare. Histopathological examination demonstrates two types of schwannomas cells: Antoni type A and Antoni type B. The Antoni type A are spindle-shaped cells with parallel rows of palisading nuclear organized in whorls and waves, while the Antoni type B consists of spindle cells haphazardly scattered in a delicate, fibrillar microcystic matrix. Most schwannomas contain a mixture of both Antoni type A and Antoni type B tissue [4, 5].

Schwannoma arose from hypoglossal nerve in the submandibular space is rare. In the present report, we described a case of schwannoma of the hypoglossal nerve in the submandibular region with imaging and histopathological findings.

## 2 CASE REPORT

A 31- years- old woman was aware of swelling at the left submandibular region in 2008, but she was left untreated because of the absence of symptoms. She was diagnosed with chronic submandibular sialoadenitis by CT at an ear, nose and throat hospital in 2013. She presented to our hospital in May 2013 for follow-up. Physical examination revealed a palpable firm, rubbery, freely mobile mass measuring about 25mm at its widest diameter in the right submandibular region. Slight tenderness was present on palpation and salivary flow was normal. The tip of the tongue was deviated to the affected side on protrusion. Routine hematologic investigation findings were normal.

**Fig. (1):**
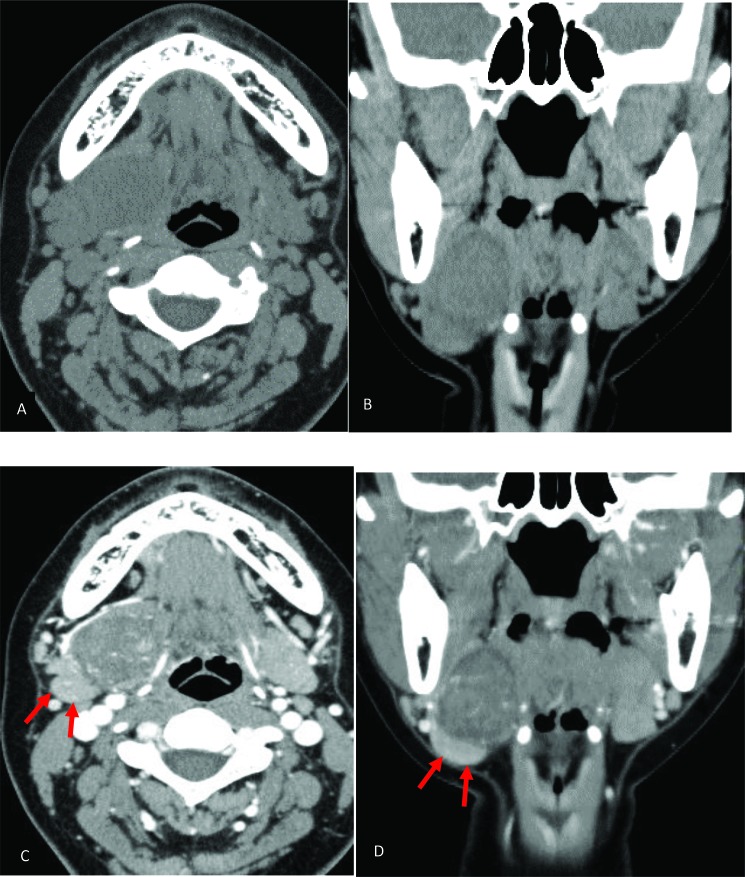
Non-contrast (A, B) and contrast-enhanced (C, D) CT images. The mass was located lateral to the genioglossus and hypoglossus muscles and the submandibular gland was compressed and inferiorly displaced (arrow). Contrast-enhanced CT demonstrated enhancement of intratumoral blood vessels.

An initial panoramic radiograph and anterior standard mandibular occlusal plain radiographs revealed nil abnormalities. Non-contrast CT revealed a well-circumscribed spherical 29 x 30 x 30 mm mass with heterogeneous density in the right submandibular region. The mass was located lateral to the genioglossus and hypoglossus muscles and the submandibular gland was compressed and inferiorly displaced. Contrast-enhanced CT demonstrated inhomogeneous weak enhancement, and intratumoral blood vessels were strong enhancement. No evidence of cystic or necrotic degeneration was present in the CT (Fig. **1**).

**Fig. (2):**
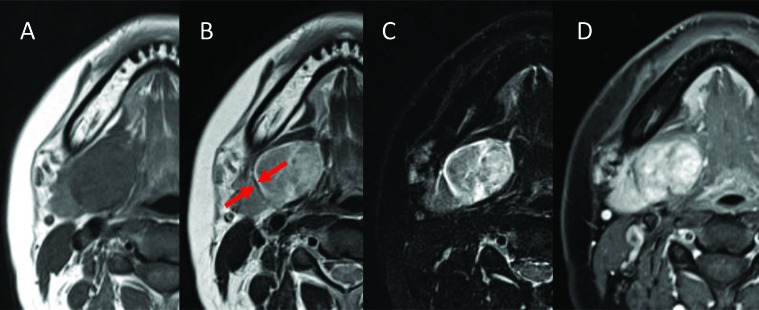
MR images showed homogeneous isointensity on T1-WI (A), heterogeneous hypointensity on T2-WI (B), heterogeneous hyperintensity on STIR (C), and heterogeneous enhancement on contrast-enhanced T1-WI (D). A clear capsule was observed on the margin (arrow).

**Fig. (3):**
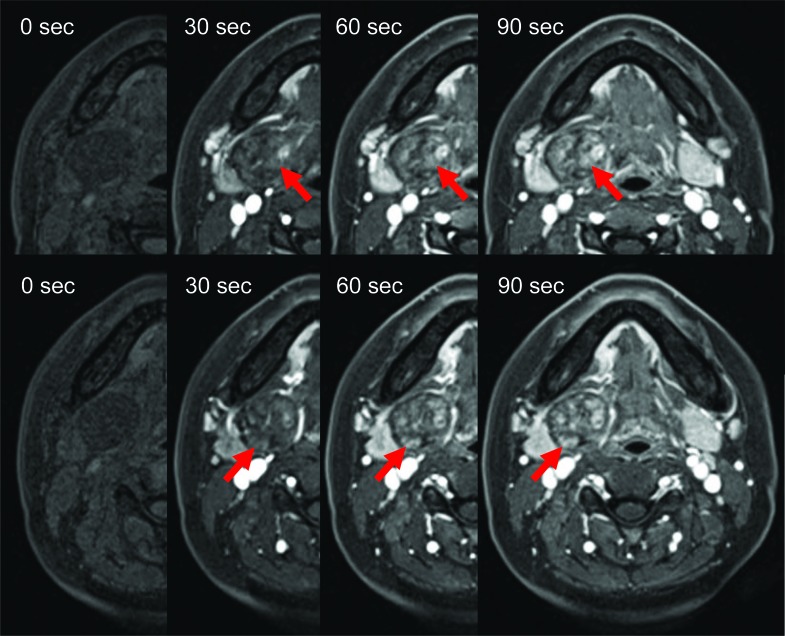
Dynamic MRI showed heterogeneous gradual increased enhancement. Regions of gradual increased enhancement were observed (red arrows).

Two weeks later, the patient underwent MRI. MR images showed homogeneous isointensity on T1-weighted imaging (T1-WI), heterogeneous hypointensity on T2-WI, heterogeneous hyperintensity on short T1 inversion recovery (STIR), and heterogeneous enhancement on contrast-enhanced T1-WI. A clear capsule was observed on the margin and showed hypointense on T2-WI. The submandibular gland was compressed and displaced inferiorly (Fig. **2**). For dynamic MRI, three-dimensional volumetric interpolated breath-hold examination shooting sequence was used with a slice thickness of 3mm. Four shots were taken at 30-seconds intervals. Dynamic MRI showed heterogeneous gradual increased enhancement. The uptake of contrast medium was regionally slow (Fig. **3**). The lesion was suspected of salivary gland tumor or neurogenic tumor on the basis of the above imaging findings. In consideration of frequency, a pleomorphic adenoma or a schwannoma was especially suspected clinically.

Fine-needle cytology and punch aspiration cytology were carried out for definitive diagnosis. Schwannoma was suspected because they revealed spindle-shaped cells. It was difficult to be confirmed as a schwannoma only by the enzyme antibody method using S-100 protein because leiomyoma was included as a differential diagnosis. However, tumors derived from smooth muscle and myoepithelial cells were denied because α-smooth muscle actin was negative. As a result, this lesion was therefore likely to be a schwannoma.

**Fig. (4):**
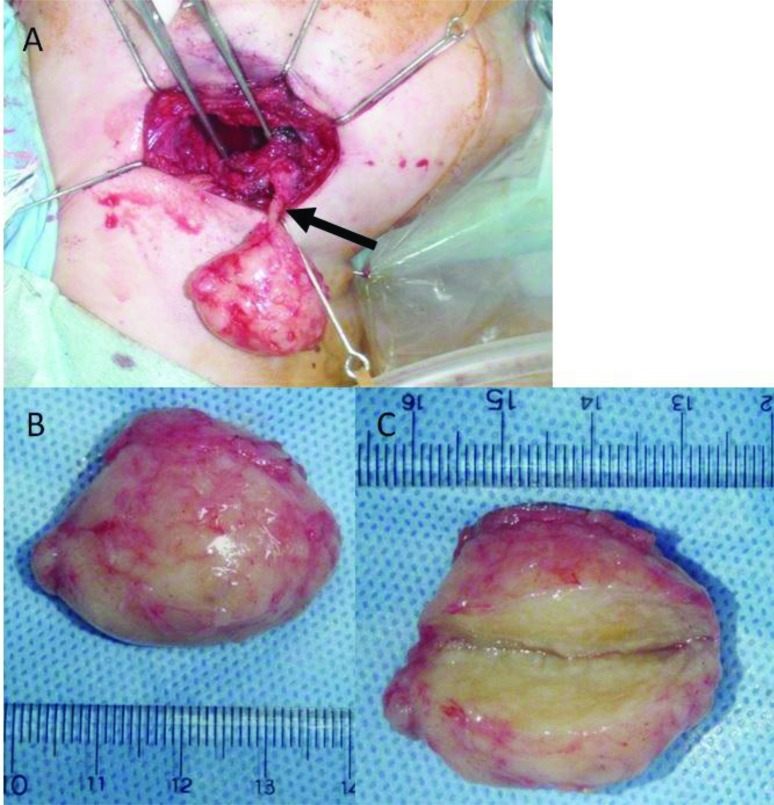
A: Intraoperatively, the tumor was well-encapsulated and continuous with the hypoglossal nerve (arrow). B, C: No cystification or necrosis degeneration was observed from the macroscopic features.

**Fig. (5):**
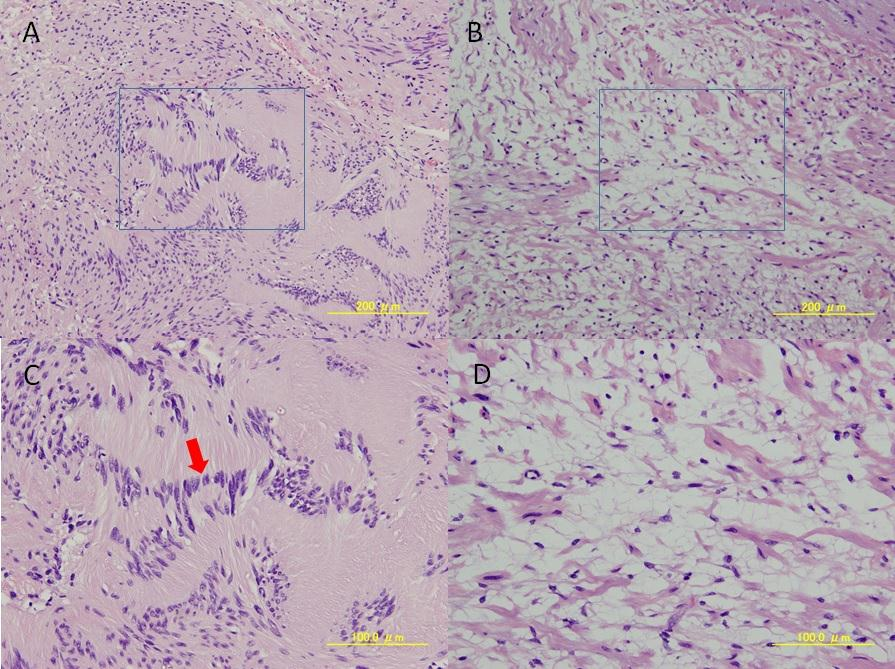
Histopathologic examination of the surgical specimen demonstrated a central mucous matrix and the surrounding nerve axon. Indicates Antoni type A of intermediate power field (A) and Antoni type B of intermediate power field (B). Each high power fields are (C) and (D). Antoni type A is composed of spindle shaped Schwann cells with elongated nuclei arranged in streams and nuclear palisades known Verocay bodies (arrow). Antoni type B consists of less spindle cells and have a myxoid background.

Total excision of the tumor was performed under general anesthesia. Intraoperatively, the tumor was well-encapsulated and continuous with the hypoglossal nerve (Fig. **4**). The tumor was excised with preservation of the submandibular salivary gland. No cystification or necrosis degeneration was observed from the macroscopic features. Histopathologic examination of the surgical specimen demonstrated a central mucous matrix and the surrounding nerve axon. The specimen also contained Antoni type A and Antoni type B (Fig. **5**). Antoni type A is composed of spindle shaped Schwann cells with elongated nuclei arranged in streams and nuclear palisades known Verocay bodies. Antoni type B consists of less spindle cells and a myxoid background. The present case contained a mixture of Antoni type A and Antoni type B; Antoni type A being the predominant microscopic pattern, alternating with Antoni type B areas occasionally. Thus, the definitive diagnosis was schwannoma of the hypoglossal nerve. In the present case, a 2-year follow-up MRI showed no evidence of recurrence.

## 3 DISCUSSION

Schwannomas are benign neoplasms of Schwann cells of the cranial, peripheral and autonomic nerves. Submandibular schwannoma involving the hypoglossal nerve is rare and presents as a non-tender, smooth swelling beneath the mucosa [1]. Except at advanced stages, pain and neurological symptoms are not evident and the overlying mucosa is not ulcerated because the tumor is smooth and encapsulated [2-6]. Schwannoma of the head and neck are most commonly located in the tongue [1, 6, 7]. Hypoglossal nerve schwannomas in the submandibular region are rare, can occur at any age and in either sex, and are usually solitary, although multiple tumors may be seen in patients with neurofibromatosis [1, 6]. Affected patients commonly exhibit deviation of the tongue towards the side of the lesion on protrusion because of damage to the infranuclear portion of the cranial nerve XII [8]. We report here a rare case of schwannoma of the hypoglossal nerve in the submandibular salivary gland region with imaging and histopathological findings.

Plain radiographs are not useful for establishing a diagnosis of schwannoma. Ultrasound examination reveals a well-circumscribed and heterogeneous mass [9]. On CT, schwannomas demonstrate low to intermediate attenuation on non-enhanced CT and variable enhancement on contrast-enhanced CT. In our case, non-contrast CT revealed a well-circumscribed spherical mass with mixed attenuation in the right submandibular region. The mass was located lateral to the genioglossus and hypoglossus muscles and the submandibular gland was compressed and inferiorly displaced. Contrast-enhanced CT demonstrated inhomogeneous weak enhancement, and intratumoral blood vessels were strong enhancement. It is reported that schwannomas tend to be cystic change due to hemorrhagic necrosis accompanying the increase of the tumor [10]. No evidence of cystic or necrotic degeneration was present. Therefore, other lesions with similar imaging characteristics include neurofibroma, leiomyoma and pleomorphic adenoma, and these should be included in the differential diagnosis.

The MRI appearances of the present case are identical to those in the previous reports [1-5, 9-11]. Schwannomas show low to intermediate signal intensity on non-enhanced T1-WI and strong enhancement with or without enhancing cystic spaces on contrast-enhanced T1-WI. On T2-WI, the tumor demonstrates heterogeneous high signal intensity and higher signal intensity than the surrounding muscles [10]. A schwannoma was strongly indicated by the heterogeneous, mottled appearance on contrast MRI, the presence of intratumoral vessels on both contrast CT and MRI, and the high attenuation and delineation of the tumoral capsule from the displaced submandibular salivary gland. Pleomorphic adenoma is common in the submandiblular space [12-14]. Both schwannoma and pleomorphic adenoma are similar in terms of exhibiting various signal intensities on T2-WI and contrast-enhanced T1-WI. Other submandibular gland lesions that were considered included submandibular lymphandenitis and cervical cyst. The MRI appearance of the tumor in the present case is identical to that in similar reports. In our case, MR images showed homogeneous isointensity on T1-WI, heterogeneous hypointensity on T2-WI, heterogeneous hyperintensity on STIR, and heterogeneous enhancement on contrast-enhanced T1-WI. A clear capsule was observed on the margin and showed hypointense on T2-WI. Then it is difficult to diagnose as a schwannoma on the basis of MR findings. The neurogenic origin of the lesion can be identified by its imaging characteristics [4]. The parent nerve is eccentric to the nerve, and the mass demonstrates split-fat, fascicular and target signs [12]. The target sign is characterized by hypointensity at the center of the mass (fibrous component) and hyperintensity at the periphery (myxomatous component) on T2-WI [9]. Unfortunately, our case didn’t include these findings. However, Dynamic MRI showed heterogeneous gradual increased enhancement. A region where uptake of contrast medium was slow was observed. Since the region of the mucinous substrate, Antoni type B, is slowly enhanced, there is a possibility that this region may reflect that. Diagnostic imaging was suspected of salivary gland tumor or neurogenic tumor. In consideration of frequency, a pleomorphic adenoma or a schwannoma was suspected.

The diagnosis of schwannoma is confirmed histopathologically by the presence of Antoni type A cells (palisading sworls of spindle cells with a central eosinophilic zone known as Verocay body), Antoni type B cells (less organized and distributed in a light fibrillar matrix), and Verocay bodies [2, 4, 12]. The present case contained a mixture of Antoni type A and Antoni type B; Antoni type A being the predominant microscopic pattern, alternating with Antoni type B are as occasionally. It was also characterized by positive immunohistochemical staining of S-100 protein and vimentin.

The treatment of choice is surgical excision with preservation of nerve function [7, 8]. Recurrence is uncommon [8]. In our case, intraoperatively, the tumor was well-encapsulated and continuous with the hypoglossal nerve. Then the present case was diagnosed as a schwannoma from the findings. Then, the 2-year follow-up MRI showed no evidence of recurrence.

## CONCLUSION

1. Histopathologic examination is inevitable, because MR findings are not specific.
2. Schwannomas were said to have specific MRI properties, including specific signs (split-fat sign, fascicular sign, target sign). However, they are not always observed.
3. This case confirmed the differential diagnosis on the basis of the intraoperative finding that the tumor was continuous with the hypoglossal nerve.
